# Facts and Observations on Diabetes

**Published:** 1817-02

**Authors:** C. W. Smerdon


*For the London Medical and Physical Journal.*

### *Facts and Observations on Diabetes;* by Mr. C. W. Smerdon.

THE Editors of a periodical publication have been pleased to copy into their publication the Case of Diabetes Mellitus which was inserted in a former Number of this Journal; and they have also thought proper to express their disapprobation of the treatment, by sagely remarking that it was quite sufficient to kill a horse. I will leave it, however, to the candour of your medical readers to decide, what part of my treatment of that case is deserving of such a sweeping conclusion. Wilson, during the progress of the disease, had been under a variety of medical men; and, no doubt, most of the known remedies had been resorted to for his cure. When I first saw him, the diabetic symptoms were mitigated by opium; but, still, it was very evident that no other benefit could be expected from this remedy than what was already obtained, and that the poor man must, at no distant period, die of the disease, unless its progress was arrested by some other more powerful means. Under all these circumstances, I think, myself perfectly justified in having recourse to a remedy which, in a number of instances, has been attended by the happiest results; and surely, having determined on the course to be pursued, it was a paramount duty, which I owed to my patient and to myself, to follow it with all the energy which the desperate nature of the case required.

But I had a still more powerful reason for trying blood-letting in this case. A gentleman, convalescent of the same disease, whose recovery was almost considered miraculous by his friends, was anxious that the same means should be tried on Wilson which had been of so much benefit to himself. This gentleman's case, although principally drawn up from his own statement, is not, I presume, devoid of interest: I have therefore sent it for insertion.

I cannot think that the present state of our knowledge warrants the conclusion that there are certain varieties of Diabetes Mellitus which are unfavourable for the bleeding plan, provided the morbid actions which constitute the disease be not altered, or rendered permanent, either by long continuance continuance or the remedies employed. If the question were asked, What circumstance occasioned the failure in Wilson's case, I would answer, principally the animal diet. This powerful remedy restrains the disease merely by the abstraction of that part of the food which the stomach, in diabetes, changes into saccharine matter; and, so long as the diseased actions remain repressed, so long will the remedy be effectual. This was precisely what happened in Wilson's case. He persevered in the animal diet, and remained tolerably well for some time; but at length the diseased actions became so strong, that, like a torrent, they bore down all opposition, and the stomach received the power of converting animal, as well as vegetable, food into sugar. Hence the inference, that, where the animal diet represses the disease for a time, but fails of giving permanent relief, the morbid actions go on in an insidious manner, until the remedy completely loses its effect, and the strength or altered state of the disease is shewn by the conversion of animal food into saccharine matter—a phenomenon with which chemistry is, I presume, wholly unacquainted. The impression on my mind therefore is, that, had Wilson conjoined, *in the first instance*, the bleeding plan with the animal diet, the most salutary effect might have been confidently expected. It is also reasonable to infer, that, as the animal diet is an unnatural one, so it must, when persevered in for some time, cause some considerable alteration or derangement in the constitution. This, I think, will, in some measure, be illustrated by the case which I shall presently relate.

One peculiarity in Wilson's case, which deserves attention, was, the apparently healthy state of his blood; whereas, in every instance, which I have heard or read of, where the remedy has proved beneficial, the blood drawn has exhibited the most unnatural* appearance.

*Case*

* This state of the blood, and the remarkably good effects which has resulted from blood-letting, have given rise to the opinion that diabetes is an inflammatory disease; but, besides the improbability that an inflammation of any internal organ should go on for a series of years without destroying it, particularly if the organ diseased be the kidney, nothing can be more dissimilar than inflamed blood, and that peculiar to diabetes. In inflammation, the crassamentum of the first-drawn blood is powerfully contracted, and, by the subsidence of the red particles, exhibits the true buffy character. After two or three evacuations of this kind, the inflammation, in the plurality of favourable cases, is subdued; and now, if

*Case of Diabetes Mellitus.*—Mr. W., an opulent manufacturer near Dublin, came here, in the summer of last year, for change of air. About twelve months previous to this, he became the subject of a severe cold, which was accompanied by unusual and rather alarming symptoms: these were, frequent acute pains about the chest and epigastrium, unpleasant dryness of the fauces, which occasioned a hoarse sound of the voice, furred tongue, great thirst, hot dry skin, confusion of idea, pain in the loins, and anasarcous swellings of the feet and legs. His urine was also increased (as was afterwards ascertained) to twelve or fourteen pints a-day; but this, in consequence of the quantity of liquid ingesta, was taken no notice of. The disease was treated as an incipient phthisis. He was desired to drink plentifully of acidulated diluents, to use frequently the warm bath, and to restrict himself to a vegetable diet.* This plan of treatment only served to increase the diabetic symptoms. The dropsical effusion of his legs became increased to an alarming degree; and, notwithstanding his appetite was voracious, he decreased rapidly in strength and flesh. The warm bath, however, produced a very salutary change on the skin, which, from being dry and scaly, became soft and perspirable, and he frequently sweated copiously whilst in the bath. Matters having gone on in this way for some months without any prospect of amendment, he was ordered into the country,

---

if venæsection be persisted in, the blood will be found attenuated, the crassamentum large, rotten (if I may so express it), convex on its surface, and partly mixed with the serum. The reverse of these states of the blood is what commonly takes place in diabetes; for here the inflammatory character does not shew itself until after three or four bleedings, whilst the first blood that is drawn is extremely attenuated, and apparently an homogeneous mass: hence the probability that the inflammatory appearance of the blood is rather the effect of the remedy, and the curative process, than of the original disease.

* Hence we learn the necessity of examining the excreta in cases where the symptoms, taken collectively, do not wholly correspond with the usual symptoms of the disease to which we are led at first to apply them.

Mr. W. was attended by the most eminent physician in Dublin; and it is but reasonable to infer, that others, equally skilful, might be deceived in a similar case, when under similar circumstances. The symptoms of diabetes, when attended by pains of the chest and swelled legs, correspond so much with those of other diseases in which there is hectic, that, unless the quantity or the quality of urine attract the attention of the medical man, it is ten to one but that he will be led into a serious error.

where where (principally by an examination of the urine, which was found to have all the diagnostic marks of diabetes mellitus) the disease was clearly defined. He was immediately put upon the animal diet, was bled copiously five times within as many weeks, and took small doses of opium, which, by a progressive increase, soon amounted to nine grains a-day. Under this plan of treatment he rapidly recovered: his urine became natural in quantity, and lost its sweetness; and he was heavier, by several pounds, at the conclusion of this treatment, than he was at the commencement of it. With the view of more quickly re-establishing his health and strength, he left Ireland for this country, and arrived at Clifton a few weeks afterwards, tolerably well, having strictly persevered in the animal diet and opium.

He had not been long here before he felt the disease creeping on him again. This was evinced by wandering acute pains about the region of the stomach and breast, which principally attacked him when asleep; by slight exacerbation of fever at night, a foul tongue, feeble quick pulse, and lassitude. His urine was not increased in quantity, but was heavy, of the colour of porter, and highly impregnated with saline matter. Three pints produced, on evaporation, four ounces and half of tenaceous matter, having the colour and consistency of thick treacle. Its smell was strongly urinous, taste not in the least sweet, but bitter,* and very salt. The slightest deviation from the animal diet was sufficient to produce a considerable change in the properties of the urine: if he ate bread for supper, along with an egg or mutton chop, an increased quantity of sweet straw-coloured urine,

---

* This bitter taste of the urine I detected also in Wilson's case, and found it only to exist when the saccharine principle of the urine was considerably decreased. Hence I drew the inference, that the bitter principle was present only when the saccharine was withdrawn, or nearly so.

I believe this morbid change of urine has not been before noticed. I am, therefore, unable to determine, if it be a common appearance and peculiar to the saccharine diabetes, or if it may be detected in the urine of other diseases. Of its nature, also, nothing can be determined upon. Proust has detected a resin in urine, which he considers the same as the resin of bile, and to which, according to him, the urine owes its colour. Wilson's was some shades darker when it contained the bitter principle than when it was sweet; and Mr. W.'s was nearly the colour of porter. If we could get over the difficulty of bile being conveyed to the kidneys or urinary bladder, and thus becoming mingled with the urine, the bitterness in question might readily be accounted for.

 for for that night, would be the consequence. All these symptoms went on increasing, until Nature set about relieving herself, by inducing copious discharges of perspiration nightly. These were preceded by general pyrexia, confusion of ideas, a quick bounding pulse, and restlessness. Towards morning, the febrile paroxysms went off, and left him, for the greater part of the day, cold and languid, with a feeble quick pulse above 100 in the minute.* At his own request, he was bled to sixteen ounces, which afforded him most decided relief. He lost the fever and night-perspirations, as well as the pains of the breast, &c.; his urine became more healthy than it had been for some time: and he considered himself as well as at any period subsequent to his first attack. Matters, however, did not long remain in this favourable state; and, at the end of a week, finding a slight return of his former symptoms, he was again bled, and with the same happy result of restoring him to a comparative state of health. Business prevented him from remaining longer at this place; but, after a lapse of six months, I was favoured with a letter from him, wherein, after complaining bitterly of the vacillating opinions of the faculty, he says, "I was bled three times after I left Clifton, and received benefit from each operation;—the crassamentum, likewise, became progressively firmer. You will wonder why I abandoned a practice from which I have twice received such considerable relief. In truth, the profession alone were to blame. They were afraid that my constitution would sink under such repeated evacuations."—After this, he consulted Dr. Bailey, who recommended him to take magnesia and laudanum daily, and iceland moss; and cheered him with the hope that the disease would give way to this treatment in the course of a few months. Experience has proved the fallacy of this. He returned to Dublin soon afterwards, where the disease became so violent, that a consultation was deemed necessary, in which it was determined to follow up Dr. Watt's plan to its fullest extent.

*Clifton, Bristol;*
*Oct.* 15, 1816.

* It is, I think, very evident that these anomalous symptoms were more the consequences of the animal diet than of the original disease. Indeed, it was this impression on the mind of Mr. W. which induced him to take bread with his usual food. He made trial of this change but once; for the effect (which has been noted) was, in his opinion, so decidedly in favour of the animal diet, that no argument could induce him to repeat it.

